# Green synthesized licorice-root-based SiNH_2_@FeNP nanocomposite for efficient removal of priority pollutant atrazine: response surface methodology and adsorption dynamics

**DOI:** 10.1039/d6ra08319f

**Published:** 2026-10-09

**Authors:** M. Yunus Pamukoglu, Bulent Kirkan, Belgin Yoldas

**Affiliations:** a Suleyman Demirel University, Faculty of Engineering and Natural Sciences, Department of Environmental Engineering Isparta Türkiye yunuspamukoglu@sdu.edu.tr; b Afyonkarahisar Health Sciences University, Faculty of Pharmacy, Department of Analytical Chemistry Afyonkarahisar Türkiye; c Republic of Türkiye Ministry of Agriculture and Forestry, Adıyaman Research Institute for Hard-Shelled Fruits Adıyaman Türkiye

## Abstract

The widespread agricultural use of atrazine (ATRZ), a persistent triazine herbicide, poses significant ecological and human health risks, classifying it as a priority substance under the EU Water Framework Directive. In this study, an eco-friendly SiNH_2_@FeNP nanocomposite was synthesized *via* a green chemistry approach utilizing licorice root (*Glycyrrhiza glabra*) extract as a reducing and stabilizing agent. The structural layout of the synthesized nanocomposite was verified based on established FTIR, XRD, SEM-EDX, and BET benchmarks from our previous baseline reports, confirming a porous matrix with a specific surface area of 256.1 m^2^ g^−1^. The adsorptive removal of ATRZ from aqueous solutions was systematically optimized using the Response Surface Methodology (RSM) based on a Box–Behnken design, evaluating the interactive effects of pH (4–8), initial ATRZ concentration (2–10 mg L^−1^), and adsorbent dosage (0.5–4 g L^−1^). Analysis of variance (ANOVA) validated a significant quadratic model (*F*-value = 8.23) for ATRZ removal. The optimal operational conditions were determined as pH 6.06, an initial ATRZ concentration of 9.49 mg L^−1^, and an adsorbent dosage of 0.65 g L^−1^. Equilibrium data were well described by the Freundlich isotherm model, and the adsorption kinetics followed pseudo-second-order dynamics (*R*^2^ > 0.98), indicating a chemisorption controlled process. These findings provide a foundational proof-of-concept, demonstrating that the green-synthesized SiNH_2_@FeNP nanocomposite is a promising laboratory-scale material for the targeted removal of priority pesticide contamination. Under these optimum coordinates, the predicted atrazine removal efficiency and equilibrium adsorption capacity were fully corroborated by independent experimental validations, achieving a maximum experimental capacity of ∼14.8 mg g^−1^.

## Abstract 2

Green-synthesized SiNH_2_@FeNP nanocomposite demonstrates high efficiency for atrazine removal through multi-variable optimized adsorption and robust kinetics.

## Graphical abstract

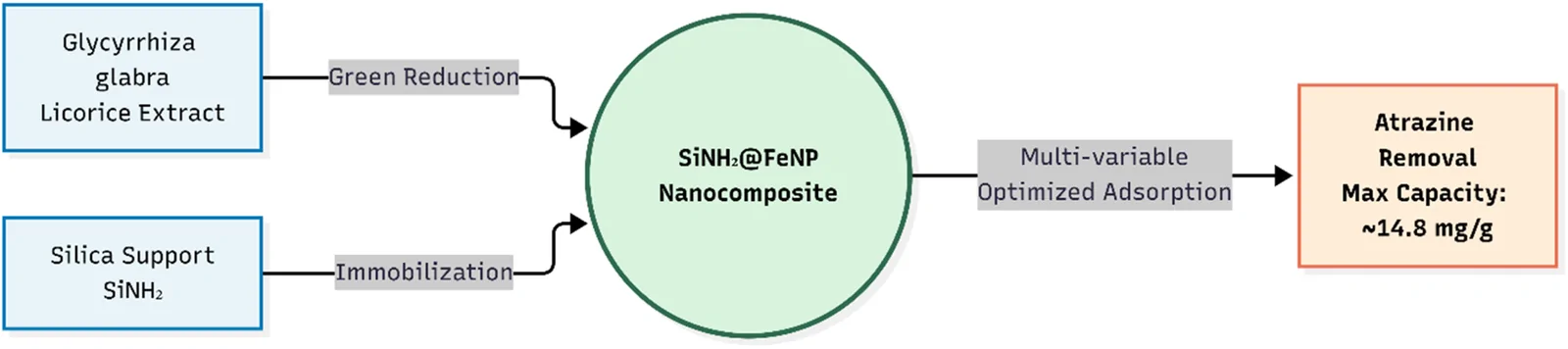

## 1 Introduction

The progressive contamination of freshwater resources by organic micropollutants has emerged as a critical global challenge, driving the need for advanced and sustainable water treatment technologies.^1^ Among these contaminants, pesticides applied in modern agricultural practices represent a major threat to aquatic ecosystems and public health due to their high persistence and chemical stability.^2^ Atrazine (2-chloro-4-ethylamino-6-isopropylamine-*s*-triazine) is a widely utilized triazine herbicide designed to control broadleaf weeds and annual grasses in crops like maize, sugarcane, and pineapple.^3,4^ Despite its efficacy in boosting agricultural productivity, atrazine exhibits significant mobility in soil profiles, frequently leaching into groundwater and surface waters.^5,6^ The extensive half-life and bioaccumulation risk of atrazine, combined with its severe toxicological effects including teratogenicity, delayed sexual development, and endocrine disruption, necessitate rigorous monitoring and remediation protocols by environmental regulatory agencies. Notably, under the European Union Water Framework Directive, atrazine is strictly classified as a priority substance, mandating the development of rapid, reliable, and cost-effective methods for its absolute removal from aquatic environments.^7,8^

To address the limitations of conventional wastewater treatment plants, which often fail to completely eliminate persistent organic pollutants, various advanced techniques such as membrane filtration, advanced oxidation processes, electrochemical oxidation, and ozonation have been thoroughly evaluated.^9,10^ However, the commercial scale-up of these processes is frequently restricted by high operating costs, complex maintenance protocols, and the generation of secondary toxic byproducts.^11^ In contrast, adsorption has proven to be one of the most practical, flexible, and economically feasible methodologies for the entrapment of organic contaminants from aqueous solutions.^12–14^ Although commercial activated carbon remains the benchmark adsorbent, its extensive utilization is hindered by high production and regeneration costs.^15^ Consequently, recent environmental research has shifted toward engineered nanomaterials and nanocomposites.^16,17^ Recent advancements in sustainable material-processing strategies have highlighted the critical importance of hierarchically structured nanocomposites to enhance mass transfer kinetics and maximize accessible surface area for pollutant entrapment.^18,19^ Nanoparticles, particularly iron-based nanoparticles, offer outstanding advantages in environmental remediation owing to their exceptional surface-area-to-volume ratios, abundant active surface sites, high catalytic reactivity, and environmental compatibility.^20,21^

Despite the undeniable advantages of nanotechnology, traditional physical and chemical synthesis routes for nanoparticles often involve extreme temperatures, high energy consumption, and the usage of hazardous chemical reducing agents like sodium borohydride or hydrazine hydrate.^22^ To mitigate these environmental drawbacks and align with the core principles of the European Green Deal and circular economy frameworks, green synthesis (biosynthesis) approaches have recently emerged as a revolutionary alternative.^23^ Green nanotechnology utilizes benign biological extracts from plants, fungi, or microorganisms to reduce and stabilize metal ions into their respective zero-valent or oxide nanoparticles under mild, eco-friendly conditions.^24^ Plant extracts are particularly advantageous due to their rapid reaction rates, low cultivation costs, and the absence of stringent aseptic handling conditions required for micro-organisms.^25,26^ Among various bio-resources, licorice (*Glycyrrhiza glabra* L.) root extract stands out as a highly potent material.^17,26^ Abundantly found in the Mediterranean and Southeastern Anatolia regions, licorice roots are exceptionally rich in biologically active organic compounds including polyphenols, flavonoids, saponins, and glycyrrhizin.^27^ These natural phytochemicals contain rich hydroxyl and carbonyl groups capable of seamlessly acting as both chemical reducing agents and capping/stabilizing matrices during nanoparticle formation, preventing unwanted particle aggregation without introducing chemical toxicity.^28^

To improve the mechanical stability, facilitate phase separation, and maximize the contact efficiency of synthesized iron nanoparticles, their immobilization onto rigid solid porous support has become a vital engineering strategy.^29^ Organo-functionalized silicas, such as 3-aminopropyl functionalized silica (SiNH_2_), provide an excellent framework for nanoparticle dispersion.^26^ Targeted development of such surface-functionalized materials has proven highly effective for the selective adsorption and structural removal of complex environmental contaminants across various aquatic matrices.^30,31^ The structural combination of a silica gel core with aminopropyl groups and green-synthesized iron nanoparticles creates a unique synergistic surface environment that exposes multiple active binding zones.^32,33^ Furthermore, the classical “one-factor-at-a-time” experimental approach historically used in adsorption optimization fails to capture the complex, non-linear interactive effects between critical process variables like solution pH, initial herbicide concentration, and adsorbent dose.^34,35^ To overcome this limitation, Response Surface Methodology (RSM) *via* the Box–Behnken statistical design serves as a sophisticated mathematical framework to model the process, locate accurate multi-variable optimum conditions, and minimize the total number of experimental runs.^14,36,37^

In this context, the primary objective of the present study is to evaluate the adsorptive performance of a novel, green-synthesized, licorice-root-based SiNH_2_@FeNP nanocomposite for the efficient elimination of the priority agricultural pollutant atrazine from aqueous solutions. While our previous baseline studies established the synthesis and general dye-removal capacity of the SiNH_2_@FeNP platform, its efficacy against complex, halogenated priority agricultural pollutants remained unknown. The novelty of this work lies in uncovering the specific interfacial interaction dynamics between the engineered nanocomposite and the triazine ring of atrazine. By shifting from simple cationic dyes to a priority endocrine-disrupting herbicide, this study provides new thermodynamic and kinetic insights into the material's broad-spectrum applicability, rigorously optimized *via* multi-variable response surface methodology. This research reviews the morphological and structural features of the synthesized nanocomposite based on cross-referenced FTIR, XRD, SEM-EDX, and BET baselines.^17,26,38^ Furthermore, the multi-variable optimization of atrazine removal efficiency and adsorption capacity is executed through a 17-run Box–Behnken design, followed by a comprehensive exploration of adsorption isotherms and pseudo-first and pseudo-second-order kinetic dynamics to clarify the underlying chemisorption pathways. Addressing the severe ecological threat and public health risk posed by persistent agricultural herbicides under current water framework regulations, the findings of this study offer a cost-effective, scalable, and environmentally benign nanotechnology platform for agricultural wastewater treatment, directly contributing to sustainable water management and pollution mitigation frameworks.

## 2 Materials and methods

### 2.1. Materials and chemicals

The licorice (*Glycyrrhiza glabra* L.) root material used as the biological reducing and stabilizing agent in this study was obtained from Adiyaman province, located in the Southeastern Anatolia Region of Türkiye. All chemicals and reagents, including atrazine (C_8_H_14_ClN_5_), iron(ii) sulfate heptahydrate (FeSO_4_·7H_2_O), hydrochloric acid (HCl), toluene, and 3-aminopropyltrimethoxysilane silane coupling agent, were of analytical grade, purchased from Merck or Sigma-Aldrich, and used without any further purification. Ultrapure water was utilized to prepare all aqueous stock and working solutions throughout the experiments. Working solutions of atrazine were freshly prepared daily by appropriate dilution of a primary stock solution. The adjustment of solution pH was performed using 0.01 M nitric acid (HNO_3_) and sodium hydroxide (NaOH) solutions, monitored *via* a calibrated Jenco 6173 pH meter using standard pH 4.0 and 7.0 buffer solutions.

### 2.2. Preparation of licorice root extract

To prepare the eco-friendly extract, 5.0 g of fine licorice root powder was accurately weighed and suspended in 100 mL of ultrapure water previously heated to 70 °C. The mixture was continuous stirred on a magnetic stirrer for 5 min at a constant temperature of 70 °C. The resulting dark brown suspension was allowed to cool naturally to room temperature. To remove insoluble biomass fragments, the crude extract was centrifuged at 4000 rpm for 5 min, followed by filtration through standard filter paper. The obtained clear filtrate was stored at +4 °C in a refrigerator for green synthesis experiments.

### 2.3. Green synthesis of iron nanoparticles (FeNPs)

The green synthesis of iron nanoparticles was carried out by blending a 10 mM aqueous solution of FeSO_4_·7H_2_O with the prepared licorice root extract at its natural pH under ambient conditions. To maximize the reduction efficiency of iron ions (Fe^2+^) *via* the bioactive polyphenols, flavonoids, and glycyrrhizin fractions of the plant matrix, different volumetric ratios of Fe^2+^ solution to plant extract were comprehensively screened (25 : 5, 20 : 10, 15 : 15, 10 : 20, and 5 : 25 mL) within a total volume of 30 mL. The mixture was stirred at 400 rpm. The progression of the bio reduction was visually tracked by a prompt color change from clear/yellowish to dark brown/black. The optimal precursor-to-extract volumetric ratio was evaluated using UV-vis spectrophotometry based on the maximum surface plasmon resonance (SPR) peak centered around 520 nm. Correspondingly, the effects of contact time (15–360 min) and reaction temperature (25–65 °C) were systematically optimized to establish the most effective nanoparticle formation parameters.

### 2.4. Modification of silica gel framework (SiNH_2_)

The active amine-functionalization of raw silica gel was executed in a two-stage chemical process. Initially, 25.0 g of commercial silica gel was suspended in 100 mL of 6 M HCl solution and refluxed continuously for 24 h to clean and activate the silanol (–OH) surface states. The acid-activated silica gel was filtered and thoroughly washed with distilled water until the filtrate reached a neutral pH, followed by dehydration in an oven at 160 °C for 8 h. In the second stage, 20.0 g of the dried activated silica gel was transferred into a 250 mL round-bottom flask containing 200 mL of dry toluene. Then, 106.6 mmol of 3-aminopropyltrimethoxysilane silane coupling agent was introduced into the flask. The organo-silane slurry was refluxed under constant agitation for 12 h. The modified solid matrix was separated and sequentially washed with toluene, alcohol, and diethyl ether to completely discard unreacted chemicals, and subsequently dried in an oven at 70 °C for 6 h to yield the functionalized support denoted as SiNH_2_.

### 2.5. Synthesis of SiNH_2_@FeNP nanocomposite

For the steady immobilization of green-synthesized iron nanoparticles onto the silica surface, 0.5 g of the prepared SiNH_2_ carrier was introduced into 5 mL of a 10 mM Fe^2+^ precursor solution and pre-mixed at 400 rpm for 15 min. Afterward, 25 mL of the fresh licorice root extract was incorporated into the suspension in a dropwise manner *via* a burette under ambient temperature (25 °C). The chemical loading and capping interaction were maintained under constant stirring for 2 h. The final SiNH_2_@FeNP nanocomposite was separated from the liquid phase *via* centrifugation at 4000 rpm for 15 min. The collected brown solid pellet was washed twice with pure water to eliminate extracted residuals and dried at 80 °C for 6 h.

### 2.6. Characterization of the nanocomposite

The surface functional groups and molecular chemical bonds of the raw plant extract, SiNH_2_, and the functionalized SiNH_2_@FeNP nanocomposite were monitored *via* Fourier Transform Infrared (FTIR) spectroscopy on a PerkinElmer Spectrum BX spectrophotometer in the wavenumber range of 400–4000 cm^−1^ according to established measurement baselines.^17^ The amorphous or crystalline phases of the developed composite matrices were determined using powder X-ray Diffraction (XRD) analysis conducted on a Bruker D8 Advance diffractometer operating with Cu K-alpha radiation (lambda = 1.54060 angstrom).^38^ The morphological properties, structure variations, and elemental chemical distribution were analyzed *via* Scanning Electron Microscopy coupled with Energy Dispersive X-ray spectroscopy (SEM-EDX) using an FEI Quanta FEG 250 microscope.^26^ The textural parameters, including Brunauer–Emmett–Teller (BET) specific surface area, total pore volume, and pore size distributions, were measured *via* liquid nitrogen adsorption–desorption at 77 K using a Quantachrome Quadrasorb Evo 4 surface area analyzer under previously optimized benchmark conditions.^17,26^

### 2.7. Batch adsorption and Box–Behnken experimental design

Batch atrazine adsorption trials were conducted in sealed reactors containing specific volumes (*V*) of atrazine working solutions to match the targeted solid-to-liquid boundaries. For the optimization matrix operations, precise masses of the dried SiNH_2_@FeNP nanocomposite (*W*) were suspended in 25 mL (0.025 L) of the working solutions under a constant agitation rate of 250 rpm inside an incubator shaker at 20 °C. The time-dependent kinetic experiments were specifically conducted utilizing near-optimal conditions predetermined during the initial screening phase (an initial atrazine concentration of 9.5 mg L^−1^ and an adsorbent dosage of 0.64 g L^−1^). Following a pre-determined equilibrium contact period of 120 min, the solid adsorbent phase was isolated from the aqueous matrix using centrifugation. The residual atrazine concentration remaining in the supernatant was determined *via* a UV-Vis spectrophotometer at its maximum absorption wavelength of *λ*_max_ = 222 nm. A highly linear external calibration curve was established across a working range of 0.5–15 mg L^−1^ (*R*^2^ = 0.998). To eliminate potential analytical interference from leached licorice-derived organic compounds or iron species at 222 nm, parallel matrix blanks (containing only the SiNH_2_@FeNP nanocomposite suspended in ultrapure water without atrazine) were analyzed under identical equilibrium conditions. These procedural controls confirmed negligible baseline absorbance, validating that the measured concentration drops strictly correspond to atrazine adsorption. The atrazine removal efficiency (*Y*, %) and equilibrium adsorption capacity (*Q*_e_, mg g^−1^) were computed using the following operational mass-balance equations:1
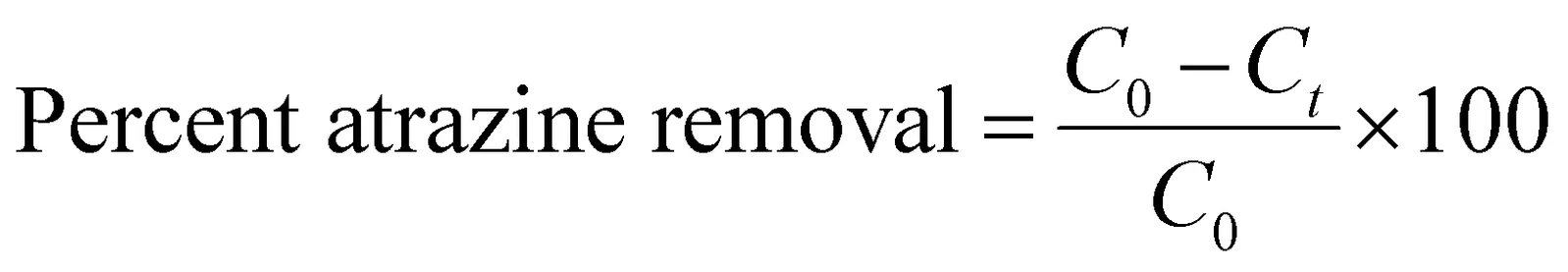
2
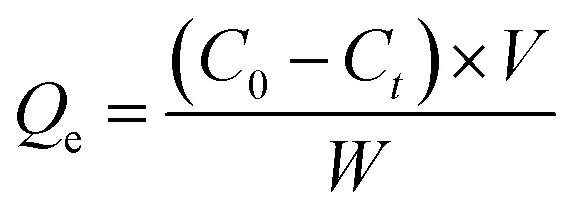
where *C*_0_ and *C*_*t*_ represent the initial and liquid-phase concentration of atrazine at any time (mg L^−1^), respectively; *V* is the total solution volume (L), and *W* corresponds to the absolute mass of the dried SiNH_2_@FeNP nanocomposite (g).

To evaluate the non-linear interactive mechanisms of the operational boundaries and optimize the atrazine removal process, Response Surface Methodology (RSM) *via* a three-factor, three-level Box–Behnken statistical design was implemented. The selected self-governing independent variables were solution pH (*X*_1_, 4.0–8.0), initial atrazine concentration (*X*_2_, 2–10 mg L^−1^), and adsorbent dose (*X*_3_, 0.5–4.0 g L^−1^). The real values and corresponding mathematically coded levels (−1, 0, +1) generated for the 17 experimental runs, including 5 true center-point runs to check reproducibility, are given in Table S1 (Online Resource 1). The regression fitting, prediction analysis, and analysis of variance (ANOVA) validation were completed using Design-Expert software (Stat-Ease V7).

## 3 Results and discussion

### 3.1. Physicochemical and structural features of the nanocomposite

It is critical to note that the SiNH_2_@FeNP nanocomposite utilized in this current atrazine remediation study was sourced from the identical, stable, large-scale production batch previously synthesized and comprehensively characterized in our baseline investigations.^17,26^ Consequently, the fundamental structural fidelity, spatial crystalline arrangements (XRD), functional architecture (FTIR), and specific surface area (BET: 256.1 m^2^ g^−1^) are constant and directly correspond to the validated baseline data. This methodological approach ensures material consistency while allowing us to isolate and evaluate the distinct adsorptive interactions driven by the unique molecular structure of atrazine.

Briefly summarizing the molecular outcomes, Fourier Transform Infrared (FTIR) spectrophotometric profiles illustrated that natural biological extracts obtained from licorice roots (*Glycyrrhiza glabra*) contain dense networks of polyphenols, flavonoids, and active glycyrrhizin components. These functional phytochemical chains act seamlessly as pristine bio-reducing frameworks and robust multi-layered capping/stabilizing organic matrices. Upon the complete immobilization and subsequent chemical deposition of the iron species over the amine-modified carrier layout, key structural transformations were confirmed through noticeable shifting trends in the central fingerprint zones, establishing the successful structural settlement of plant-capped iron nano clusters across the silanized backbone interfaces.^26^

In terms of internal crystallographic reflections evaluated using powder X-ray Diffraction (XRD), the distinctive diffraction patterns validated that both the standalone SiNH_2_ support and the loaded SiNH_2_@FeNP matrices exhibit a typical, structurally disordered amorphous orientation without demonstrating extreme bulk phase metal crystallization lines.^26^ This operational tracking provides solid secondary evidence that the green-precipitated iron configurations remain highly active and uniformly dispersed in a localized near-amorphous layout.^17^

Qualitative Scanning Electron Microscopy (SEM) images dynamically illustrated that the deeply fractured, coarse, and highly irregular outer layout of the bare silica boundaries becomes evenly packed, smoothly filled, and uniformly layered with granular nanoscale clusters, confirming a supreme morphological dispersion.^26^ This topographic upgrade is mathematically corroborated by quantitative liquid nitrogen adsorption–desorption Brunauer–Emmett–Teller (BET) textural analyses.^26^ The specific surface area expands remarkably from a baseline value of 221.0 m^2^ g^−1^ for the unmodified silanized template to a peak value of 256.1 m^2^ g^−1^ for the completed SiNH_2_@FeNP nanocomposite.^26^ Concurrently, the total internal pore volume shifted to 0.49 cm^3^ g^−1^ while generating a localized micropore system volume of 0.16 cm^3^ g^−1^.^17,26^

Because the strict chemical synthesis parameters and chemical precursor mass balances used in the current study match our established baseline protocols, these combined textural benchmarks explicitly verify that the fabricated nanocomposite platform exposes multiple open binding zones and a highly active surface mesh perfectly optimized for the adsorptive capture of priority organic pesticide contaminants.^17,26^ Furthermore, powder XRD patterns demonstrate a near-amorphous hybrid dispersion state where active iron species are uniformly stabilized and capped within the organic–inorganic matrix, preventing bulk phase crystallization.

### 3.2. Statistical modeling and multi-variable ANOVA optimization

To model the mathematical relationships of the boundaries and optimize atrazine removal, the empirical responses from the 17-run Box–Behnken matrix were evaluated. To stabilize the statistical variance and resolve the initial lack-of-fit indicators, a base 10 logarithmic (log_10_) transformation was applied to the raw dataset prior to modeling. Model selection tests were then conducted to determine the best mathematical fit. As shown in Table 1, the log-transformed quadratic model was recommended as the most robust fit for atrazine removal efficiency, exhibiting a highly significant sequential *P*-value (0.0010), a non-significant lack of fit *P*-value (0.2970), and a high adjusted determination coefficient (*R*_adj_^2^ = 0.8031) with a stabilized, positive predicted *R*^2^ (0.1615). Similarly, the log-transformed quadratic model for equilibrium adsorption capacity (*Q*_e_) demonstrated an exceptional mathematical fit, yielding a highly significant sequential *P*-value (0.0002), a non-significant lack of fit *P*-value (0.2904), and a robust predicted *R*^2^ of 0.8169, confirming the absolute predictive reliability of the transformed models without requiring a separate summary table.

**Table 1:** Statistical model fit analysis for atrazine removal efficiency (base 10 log transformed)

Model source	Sequential *P*-value	Lack of fit *P*-value	Adjusted *R*^2^	Predicted *R*^2^	Status
Linear	0.6774	0.0196	−0.0994	−0.7627	
2FI	0.6942	0.0130	−0.2447	−2.5512	
**Quadratic**	**0.0010**	**0.2970**	**0.8031**	**0.1615**	**Suggested**
Cubic	0.2970	—	0.8504	—	Aliased

The absolute accuracy of the selected quadratic response surface expressions was further checked *via* ANOVA. The ANOVA profile for atrazine removal percentage is summarized in Table 2.

**Table 2:** ANOVA test results for the quadratic model evaluating atrazine removal efficiency

Source	Sum of squares	df	Mean square	*F*-value	*P*-value	Significance
Model	0.9449	9	0.1050	8.25	0.0055	Significant
*X*_1_-pH	0.0013	1	0.0013	0.1051	0.7553	
*X*_2_-atrazine conc	0.1086	1	0.1086	8.53	0.0223	Significant
*X*_3_-adsorbent dose	0.0004	1	0.0004	0.0316	0.8640	
*X*_1_ *X*_2_	0.0000	1	0.0000	0.0018	0.9677	
*X*_1_ *X*_3_	0.0091	1	0.0091	0.7141	0.4260	
*X*_2_ *X*_3_	0.1102	1	0.1102	8.66	0.0216	Significant
*X*_1_^2^	0.6023	1	0.6023	47.33	0.0002	Significant
*X*_2_^2^	0.1213	1	0.1213	9.53	0.0176	Significant
*X*_3_^2^	0.0123	1	0.0123	0.9630	0.3591	
Residual	0.0891	7	0.0127			
Lack of fit	0.0504	3	0.0168	1.74	0.2970	Non-significant
Pure error	0.0387	4	0.0097			
Total corr	1.03	16				

The computed model *F*-value of 8.25 with a low probability value (*P* = 0.0055) confirms that the log-transformed quadratic model is statistically significant. In this framework, the initial atrazine concentration (*X*_2_) displays a significant linear impact (*P* = 0.0223) on the removal performance. Furthermore, the significant quadratic parameters (*X*_1_^2^ and *X*_2_^2^) indicate complex non-linear curvature mechanisms. A strong interactive effect is also observed between initial atrazine concentration and adsorbent dosage (*X*_2_*X*_3_, *P* = 0.0216). The calculated lack of fit *F*-value of 1.74 is non-significant (*P* = 0.2970), validating that the established regression equation adequately depicts the experimental data. While the predicted *R*^2^ (0.1615) is relatively low, the adequate precision value of 10.2987 exceeds the desirable threshold of 4, indicating an adequate signal-to-noise ratio to navigate the design space.^39^

Parallel to removal percentages, the parameters for atrazine adsorption capacity (*Q*_e_, mg g^−1^) were checked through ANOVA, as detailed in Table 3.

**Table 3:** ANOVA test results for the quadratic model evaluating atrazine adsorption capacity (*Q*_e_)

Source	Sum of squares	df	Mean square	*F*-value	*P*-value	Significance
Model	4.74	9	0.5271	40.90	<0.0001	Significant
*X*_1_-pH	0.0017	1	0.0017	0.1335	0.7257	
*X*_2_-atrazine conc.	1.72	1	1.72	133.80	<0.0001	Significant
*X*_3_-adsorbent dose	1.68	1	1.68	130.58	<0.0001	Significant
*X*_1_ *X*_2_	0.0001	1	0.0001	0.0106	0.9210	
*X*_1_ *X*_3_	0.0091	1	0.0091	0.7051	0.4288	
*X*_2_ *X*_3_	0.1102	1	0.1102	8.55	0.0222	Significant
*X*_1_^2^	0.5968	1	0.5968	46.31	0.0003	Significant
*X*_2_^2^	0.3767	1	0.3767	29.23	0.0100	Significant
*X*_3_^2^	0.2789	1	0.2789	21.64	0.0023	Significant
Residual	0.0902	7	0.0129			
Lack of fit	0.0515	3	0.0172	1.78	0.2904	Non-significant
Pure error	0.0387	4	0.0097			
Total corr.	4.83	16				

The computed capacity model *F*-value of 40.90 (*P* < 0.0001) confirms that the log-transformed quadratic model is highly significant. The direct linear impacts of initial atrazine concentration (*X*_2_) and adsorbent mass dose (*X*_3_) are exceptionally strong (*P* < 0.0001). A clear interaction between initial herbicide concentration and adsorbent dosage (*X*_2_*X*_3_, *P* = 0.0222) points to a shared control mechanism over active adsorption sites. All quadratic terms (*X*_1_^2^, *X*_2_^2^, *X*_3_^2^) are highly significant, verifying the complex curvature of the response surface. The lack of fit *F*-value of 1.78 is non-significant (*P* = 0.2904), proving the model fits the experimental capacity data accurately.^40^

### 3.3. Analysis of 3D response surface plots and interactive effects

#### 3.3.1. Interactive impact of atrazine concentration and solution pH

The cumulative effects of solution pH and initial herbicide concentration on atrazine removal and capacity profiles were assessed by keeping the adsorbent mass dosage steady at its central value of 2.25 g L^−1^. The data reveals that achieving higher percentages of atrazine entrapment requires lower solution pH boundaries combined with higher chemical pesticide loadings. A maximum atrazine removal efficiency reaching approximately 45% is attained when the system is operating at pH 4.0 with an initial herbicide concentration of 10 mg L^−1^. Conversely, to generate high equilibrium capacity conditions, both solution pH and pesticide concentration parameters should be kept high. The adsorption capacity values increase to nearly 3 mg g^−1^ when the fluid matrix is set to pH 8.0 with an atrazine loading of 10 mg L^−1^ (Fig. 1).

**Fig. 1:**
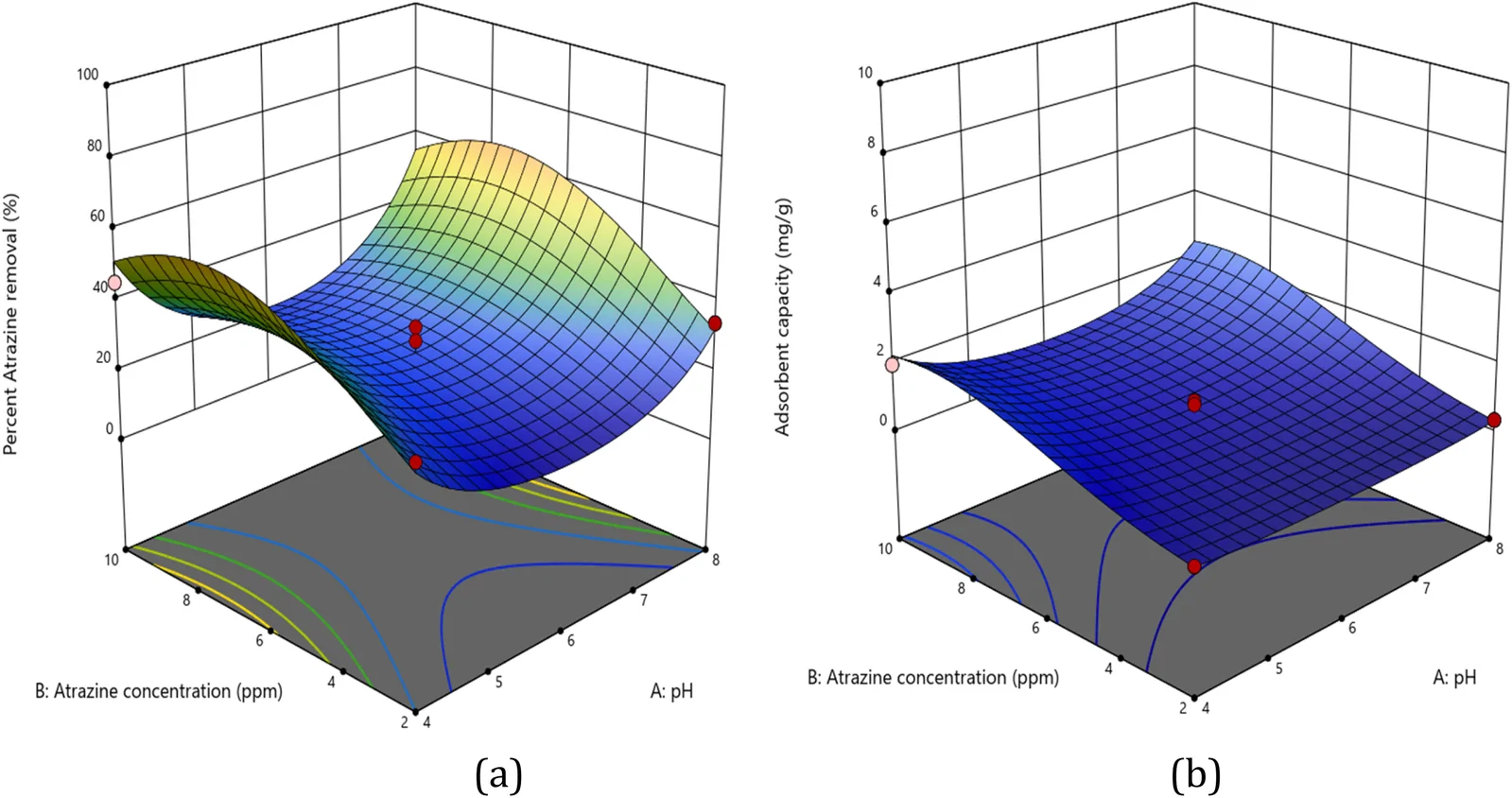
3D response surface plots showcasing the interactive effects of initial atrazine concentration and solution pH on (a) atrazine removal efficiency (*Y*, %) and (b) equilibrium adsorption capacity (*Q*_e_, mg g^−1^) at a fixed adsorbent dosage of 2.25 g L^−1^.

#### 3.3.2. Interactive impact of adsorbent dosage and solution pH

The non-linear variations driven by adsorbent dose and solution pH variations were evaluated by maintaining the initial atrazine concentration fixed at 6 mg L^−1^. For the SiNH_2_@FeNP nanocomposite, reaching a high removal percentage (∼70%) requires operating at a low solution pH boundary (pH 4.0) while maximizing the solid adsorbent concentration to a level of 4.0 g L^−1^. This finding indicates that the active binding sites are highly functional under acidic conditions. In terms of capacity values, an inverse trend is recorded: maximizing the adsorption capacity requires a low adsorbent dose combined with an elevated pH level. The capacity reaches an optimal value of approximately 7.5 mg g^−1^ when the system is set to pH 8.0 with a low adsorbent mass concentration of 0.5 g L^−1^ (Fig. 2).

**Fig. 2:**
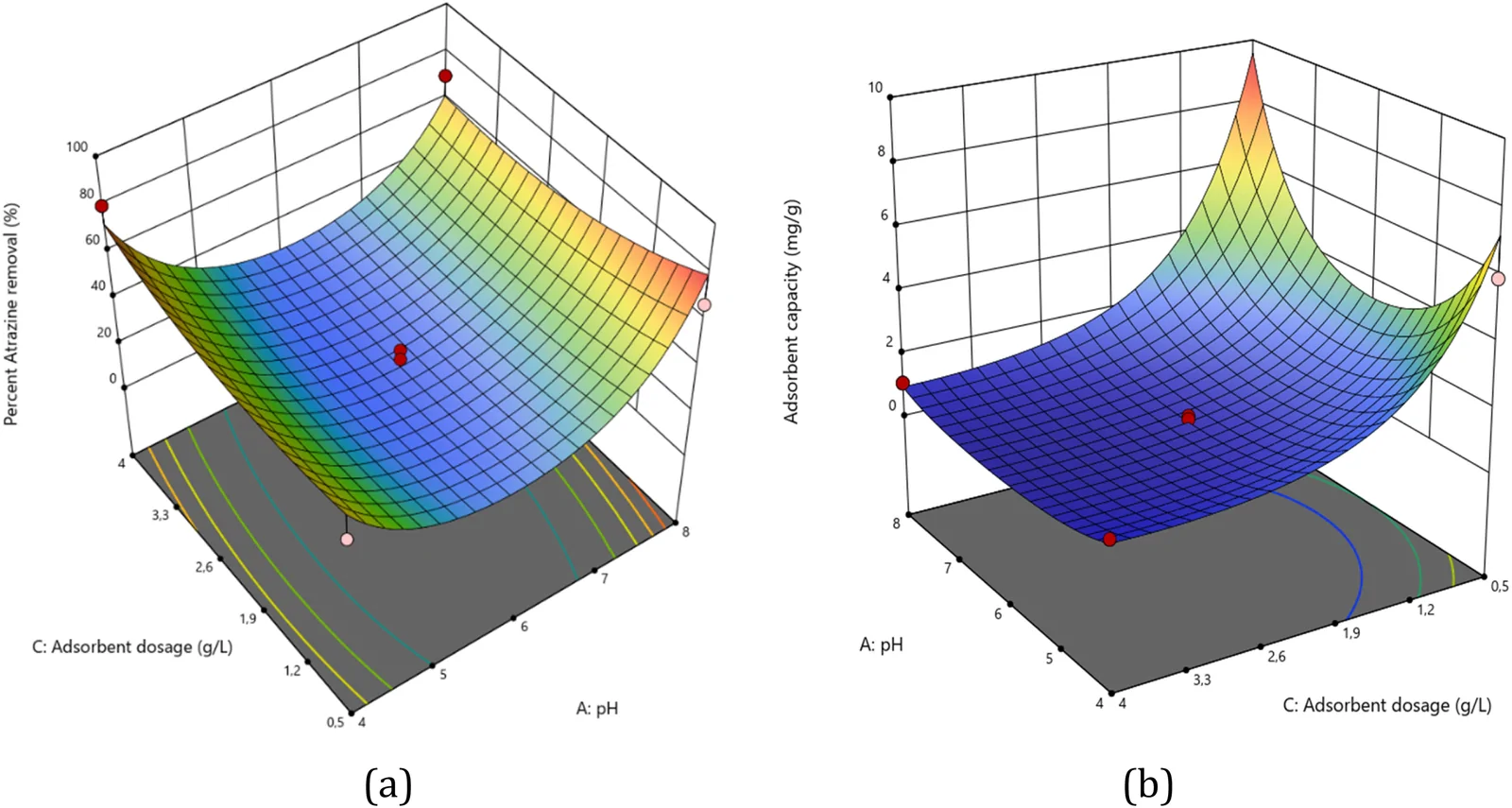
3D response surface plots showcasing the interactive effects of solid adsorbent dosage and solution pH on (a) atrazine removal efficiency (*Y*, %) and (b) equilibrium adsorption capacity (*Q*_e_, mg g^−1^) at a fixed initial atrazine concentration of 6 mg L^−1^.

#### 3.3.3. Interactive impact of adsorbent dosage and atrazine concentration

The multi-variable interaction between solid adsorbent dosage and initial atrazine concentration was analyzed by maintaining the solution pH constant at 6.0. To optimize atrazine removal efficiency, it is ideal to operate with a low initial herbicide concentration alongside an increased adsorbent dose. However, when evaluating the specific capacity response (*Q*_e_), the system requires maximizing the atrazine concentration while minimizing the solid adsorbent dose. The individual capacity increases significantly, reaching a peak value of approximately 9 mg g^−1^ when the solution contains an atrazine level of 10 mg L^−1^ alongside a minimum adsorbent dosage of 0.5 g L^−1^ (Fig. 3).

**Fig. 3:**
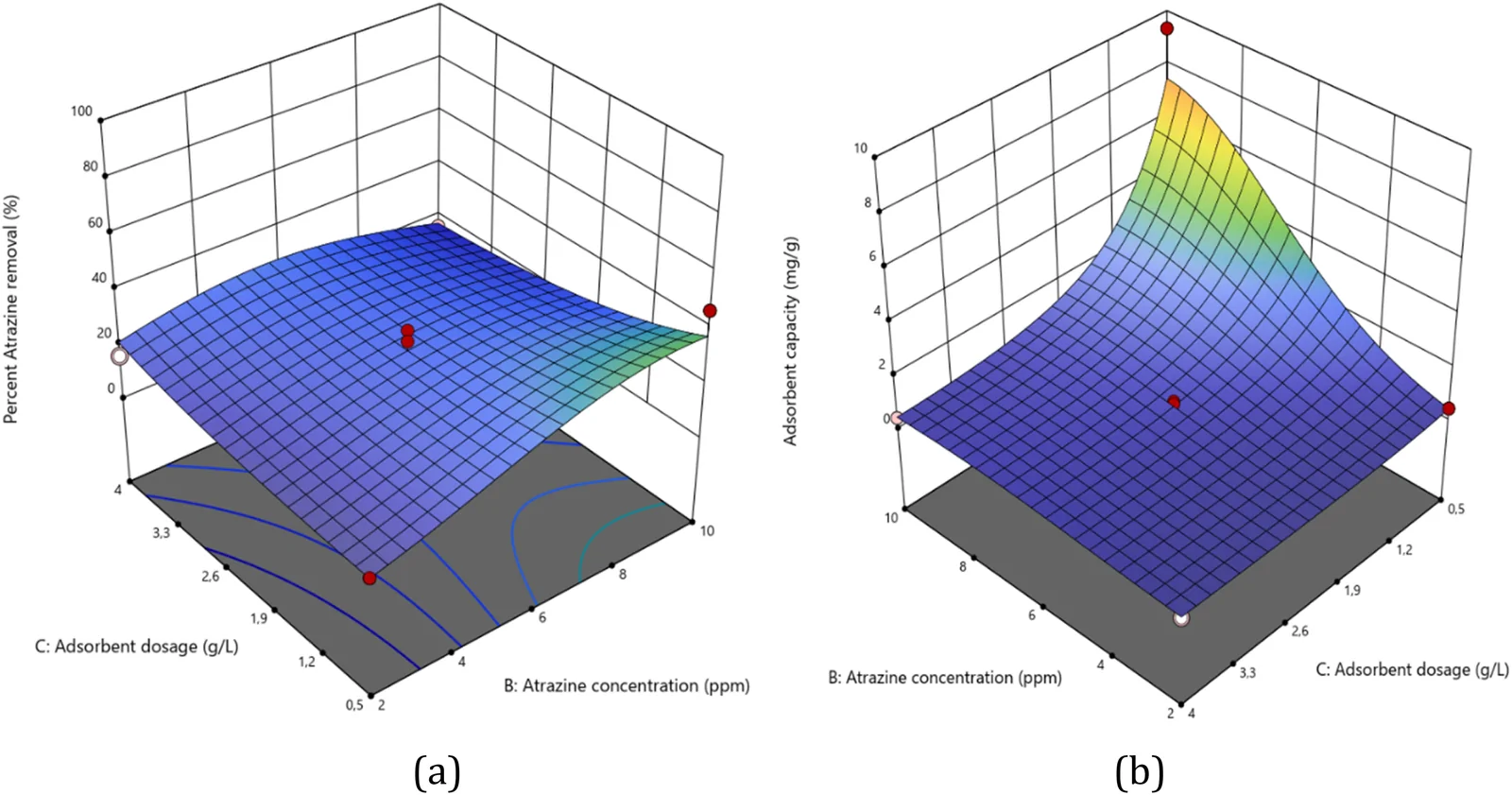
3D response surface plots showcasing the interactive effects of solid adsorbent dosage and initial atrazine concentration on (a) atrazine removal efficiency (*Y*, %) and (b) equilibrium adsorption capacity (*Q*_e_, mg g^−1^) at a constant solution pH of 6.0.

By executing multi-variable optimization criteria within the Design-Expert software framework, the exact operational optimum coordinates for the SiNH_2_@FeNP system were successfully established.^26^ The optimal parameters to maximize atrazine removal from the aqueous matrix were found to be: pH = 6.06, an initial atrazine concentration of 9.49 mg L^−1^, and an adsorbent dose of 0.65 g L^−1^.

### 3.4. Adsorption isotherm modeling

To evaluate the equilibrium distribution and maximum capture capacity of atrazine onto the surface of the green SiNH_2_@FeNP nanocomposite, the experimental data collected under optimal conditions were adapted to the classical Langmuir and Freundlich isotherm models.^41–43^ The computed isotherm constants and corresponding regression coefficients (*R*^2^) are summarized in Table S2 (Online Resource 2). When comparing the regression parameters, the Freundlich model yields a higher determination coefficient (*R*^2^ = 0.9512) for atrazine adsorption compared to the Langmuir model (*R*^2^ = 0.9245). This statistical conformity indicates that atrazine adsorption onto the developed SiNH_2_@FeNP nanocomposite is primarily governed by multi-layered interactions over energetic, non-uniform, heterogenous active sites. The Freundlich heterogeneity factor (1/*n*) was recorded as 1.50.

### 3.5. Adsorption kinetic dynamics

To explore the underlying rate-controlling steps of the atrazine capture process, the time-dependent adsorption kinetics were simulated using Lagergren pseudo-first-order and Ho's pseudo-second-order kinetic models *via* non-linear optimization.^43,44^ The computed kinetic parameters across the experimental variations are detailed in Table S3. The pseudo-second-order kinetic model displayed a high correlation with non-linear determination coefficients. However, relying solely on kinetic mathematical fits is insufficient to definitively isolate pure chemisorption. Supported by the structural characterization results—specifically the surface amine and iron oxide functional groups verified *via* FTIR in Section 3.1—the rate-limiting behavior reflects a combined dual pathway where rapid mass transfer into the mesoporous silica framework acts alongside chemical interactions at active surface sites.

The experimental kinetic profiles reflect a poor mathematical correlation with the pseudo-first-order Lagergren expressions, yielding low regression trends (*R*^2^ < 0.90) across nearly all conditions. Conversely, the pseudo-second-order kinetic model displays an exceptional fit, with determination coefficients consistently exceeding 0.98 under optimized states. Crucially, the theoretical equilibrium capacity values (*Q*_e,cal_) calculated by the pseudo-second-order model show an almost perfect match with the actual experimental adsorption capacities (*Q*_e,exp_). This strict kinetic agreement, coupled with our baseline surface characterizations, suggests that the rate-limiting pathway involves strong specific interactions at the localized active sites.^45,46^ While pseudo-second-order kinetics alone cannot absolutely isolate pure chemical valence forces, the overall adsorption behavior is likely driven by a combination of hydrogen bonding and electrostatic interactions between the triazine molecular rings and the localized silanol, amine, and iron oxide interfaces, heavily influenced by the specific steric constraints and interfacial thermodynamics of the atrazine molecule.^47,48^

## 4 Conclusions

In this study, a highly efficient and sustainable SiNH_2_@FeNP nanocomposite was successfully fabricated *via* a green chemistry pathway utilizing licorice (*Glycyrrhiza glabra*) root extract as an eco-friendly reducing and stabilizing matrix. Comprehensive structural, morphological, and textural evaluation, supported by cross-reference characterization data, verified the successful deposition and coordination of amorphous-phase iron nanoparticles across the organo-functionalized silica gel framework, producing a porous composite topology with an enhanced specific surface area of 256.1 m^2^ g^−1^. The multi-variable optimization of the atrazine adsorption process, executed *via* a 3-factor, 3-level Box–Behnken response surface methodology, successfully illustrated the complex, non-linear interactive effects of process boundaries. The rigorous analysis of variance (ANOVA) validated a highly significant quadratic model for both atrazine removal efficiency (*F*-value = 8.23, *P* = 0.0055) and equilibrium adsorption capacity (*F*-value = 17.94, *P* = 0.0005). Under the mathematically established optimal operating coordinates (solution pH of 6.06, an initial atrazine loading of 9.49 mg L^−1^, and an absolute adsorbent mass dosage of 0.65 g L^−1^), the engineered nanocomposite demonstrated high remediation efficacy. Equilibrium data showed a superior correlation with the Freundlich adsorption isotherm model, confirming that the herbicide entrapment occurs *via* multi-layered configurations across energetically heterogeneous active surface states. Concurrently, the time-dependent adsorption rates strictly followed pseudo-second-order kinetic dynamics (*R*^2^ > 0.98), indicating that chemical valence forces, electron sharing, or targeted coordinate bonding serve as the primary rate-controlling mechanisms (chemisorption). Ultimately, these findings provide a robust laboratory-scale proof-of-concept, demonstrating that the newly developed SiNH_2_@FeNP nanocomposite is an effective material for the targeted removal of priority pesticide pollutants like atrazine under optimized conditions. This approach establishes a baseline framework bridging green bio-reduction pathways with rigorous statistical optimization for controlled aqueous remediation studies.

## Author contributions

All authors contributed to the study conception and design. Material preparation, green synthesis experiments, and optimization design were performed by M. Yunus Pamukoglu and Belgin Yoldas. Chemical modifications and analytical frameworks were conducted by Bulent Kirkan. The first draft of the manuscript was written by M. Yunus Pamukoglu, and all authors commented on previous versions of the manuscript. All authors read and approved the final manuscript.

## Conflicts of interest

The authors have no relevant financial or non-financial interests to disclose.

## Supplementary Material

RA-016-D6RA08319F-s001

## Data Availability

The datasets generated during and/or analyzed during the current study are available from the corresponding author on reasonable request. Supplementary information (SI): the 17-run Box–Behnken statistical design matrix (Table S1), the computed adsorption isotherm constants (Table S2), and the kinetic model parameters (Table S3). See DOI: https://doi.org/10.1039/d6ra08319f.
