# Supplementary material for: Green synthesized licorice-root-based SiNH2@FeNP nanocomposite for efficient removal of priority pollutant atrazine: response surface methodology and adsorption dynamics
Source: RSC Adv. 2026 Oct 9;16(47):61950–8. doi: 10.1039/d6ra08319f (PMC13651744; doi:10.1039/d6ra08319f)
Supplement: Supplementary file 1 [file RA-016-D6RA08319F-s001.pdf]

Supplementary Information (SI) for RSC Advances.
This journal is © The Royal Society of Chemistry 2026

Supplementary Information

Table S1. Real and coded independent variable levels used in the Box-Behnken experimental design for atrazine adsorption

**Run No**	**pH (X1)**	**Atrazine Conc. (X2, mg/L)**	**Adsorbent Dose (X3, g/L)**
**1**	4 (-1)	10 (+1)	2.25 (0)
**2**	4 (-1)	6 (0)	0.5 (-1)
**3**	8 (+1)	6 (0)	4 (+1)
**4**	6 (0)	6 (0)	2.25 (0)
**5**	8 (+1)	2 (-1)	2.25 (0)
**6**	6 (0)	2 (-1)	4 (+1)
**7**	4 (-1)	2 (-1)	2.25 (0)
**8**	8 (+1)	10 (+1)	2.25 (0)
**9**	6 (0)	6 (0)	2.25 (0)
**10**	6 (0)	2 (-1)	0.5 (-1)
**11**	6 (0)	6 (0)	2.25 (0)
**12**	4 (-1)	6 (0)	4 (+1)
**13**	6 (0)	10 (+1)	0.5 (-1)
**14**	6 (0)	6 (0)	2.25 (0)
**15**	6 (0)	10 (+1)	4 (+1)
**16**	8 (+1)	6 (0)	0.5 (-1)
**17**	6 (0)	6 (0)	2.25 (0)

Table S2. Langmuir and Freundlich adsorption isotherm parameters for atrazine removal

Isotherm Model	Parameter Component	Calculated Value (Non-Linear Fit)
**Langmuir**	q_{max} (mg/g)	12.85
	K_L (L/mg)	0.45
	R^2	0.9245
**Freundlich**	K_F ((mg/g)(L/mg)$^{1/n}$)	3.12
	$1/n$	0.58
	R^2	0.9512

Table S3. Statistical kinetic model constants calculated for atrazine adsorption onto $SiNH_2$@FeNP

Boundary Condition Type	Pseudo-First-Order Model (R^2)	Pseudo-Second-Order Model (R^2)	q_e,exp (mg/g)	q_e,cal (mg/g)	k_2 (g/mg.min)
**pH Effects**					
*** pH 4.0**	0.7907	0.9284	5.15	5.88	0.0173
*** pH 5.0**	0.7314	0.8998	5.43	6.43	0.0118
*** pH 6.0**	0.8610	0.9817	6.42	6.95	0.0268
*** pH 7.0**	0.8487	0.9962	8.40	8.68	0.0454
*** pH 8.0**	0.7541	0.9829	9.94	10.53	0.0192
**Concentration Effects**					
*** 2 mg/L**	0.9106	0.9990	1.54	1.55	0.6185
*** 4 mg/L**	0.8695	0.9792	3.08	3.33	0.0497
*** 6 mg/L**	0.8003	0.9856	4.55	4.74	0.0476
*** 8 mg/L**	0.8025	0.9951	5.76	5.93	0.0557
*** 10 mg/L**	0.7963	0.9890	7.19	7.50	0.0352
**Adsorbent Dosage Effects**					
*** 0.5 g/L**	0.8081	0.9993	8.35	8.40	0.1156
*** 1.0 g/L**	0.8842	0.9975	4.08	4.14	0.1338
*** 2.0 g/L**	0.9791	0.9920	1.77	1.86	0.1557
*** 3.0 g/L**	0.8638	0.9896	0.76	0.76	0.5022
*** 4.0 g/L**	0.8108	0.9954	0.27	0.27	1.4935

*: Repetitive independent batch operations were conducted by holding the non-targeted operational variables constant near the optimized boundaries (pH = 6.0, initial concentration = 9.5 mg/L, or adsorbent dosage = 0.64 g/L) within a total boundary period of 120 min.
